# Detection of Micro-Cracks in Metals Using Modulation of PZT-Induced Lamb Waves

**DOI:** 10.3390/ma13173823

**Published:** 2020-08-29

**Authors:** Sang Eon Lee, Jung-Wuk Hong

**Affiliations:** Department of Civil and Environmental Engineering, Korea Advanced Institute of Science and Technology, Daejeon 34141, Korea; sangeon1@kaist.ac.kr

**Keywords:** nonlinear modulation, defects diagnosis, fatigue crack, ultrasonics, numerical simulation

## Abstract

The ultrasonic modulation technique, developed by inspecting the nonlinearity from the interactions of crack surfaces, has been considered very effective in detecting fatigue cracks in the early stage of the crack development due to its high sensitivity. The wave modulation is the frequency shift of a wave passing through a crack and does not occur in intact specimens. Various parameters affect the modulation of the wave, but quantitative analysis for each variable has not been comprehensively conducted due to the complicated interaction of irregular crack surfaces. In this study, specimens with a constant crack width are manufactured, and the effects of various excitation parameters on modulated wave generation are analyzed. Based on the analysis, an effective crack detection algorithm is proposed and verified by applying the algorithm to fatigue cracks. For the quantitative analysis, tests are repeatedly conducted by varying parameters. As a result, the excitation intensity shows a strong linear relationship with the amount of modulated waves, and the increase of modulated wave is expected as crack length increases. However, the change in the dynamic characteristics of the specimen with the crack length is more dominant in the results. The excitation frequency is the most dominant variable to generate the modulated waves, but a direct correlation is not observed as it is difficult to measure the interaction of crack surfaces. A numerical analysis technique is developed to accurately simulate the movement and interaction of the crack surface. The crack detection algorithm, improved by using the observations from the quantitative analyses, can distinguish the occurrence of modulated waves from the ambient noises, and the state of the specimens is determined by using two nonlinear indexes.

## 1. Introduction

Fatigue and corrosion are two of the main causes of metal structure failure [1]. The structure can withstand loads lower than the yield stress, but fatigue failure occurs if the structure is exposed to loads repeatedly. A fatigue crack easily initiates at the weak points where stress concentration occurs by the geometrical characteristics. Monitoring of such points, and crack detection when they first initiate, are very important tasks needed to prolong the structure lifespans, as crack growth speed gradually increases with time [2].

Various non-destructive testing methods—visual testing, penetrant testing, eddy current testing, radiographic testing, thermography, and ultrasonic testing—have been used to detect cracks [3,4], with the particular application depending on crack size, type, and location [5,6]. Ultrasonic testing is a non-destructive testing technique, which uses ultrasonic wave propagation in an object to test its integrity. Ultrasonic testing methods are popularly applied to metals and alloys, while at a lower resolution, they can also be used on concrete, wood, and composites. This method has the advantage of being able to detect defects inside objects, compared to other methods, and includes both linear and nonlinear methods.

The linear ultrasonic technique uses the reflection or refraction that occurs during a sound wave passing through a crack [4]. Defects cause reflections and refractions to the wave, due to differences in acoustic impedance. The time of flight of the propagating wave can be predicted using the material properties of the specimen, and the length of wave travel. The arrival time of reflected or refracted waves varies from that of unaffected waves, due to the different propagation path, and by analyzing the measured signals at the sensing point, the existence of defects and the approximate position of any crack can be calculated. Nonlinear ultrasonic test methods have been continuously developed, and are used to detect micro-cracks with high sensitivity [7]. With this method, cracks are detected by using nonlinear wave properties generated by the interaction of crack surfaces—harmonics, subharmonics, wave modulation, and resonance frequency shifts. In general, the nonlinear ultrasonic method observes signals in the frequency domain through the process of decomposing a function of time into frequency components [8].

**Figure 1:**
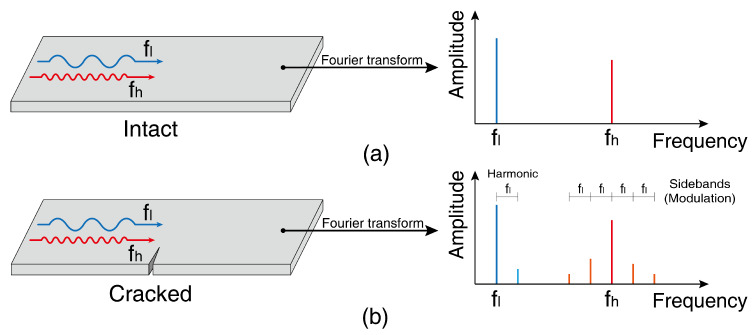
(**a**) Linear waves and (**b**) nonlinear waves in the frequency domain.

A Fourier-transformed function consists of frequency components in a complex domain. The absolute value represents the magnitude of the frequency component, and the phase angle represents the phase offset. To apply the nonlinear method, it is necessary to remove other nonlinearity-generating elements besides defects, such as surface coatings, loose bolting, and non-welded contact surfaces. In the workplace environment, these factors make crack detection using modulated wave generation complex. Among those nonlinear testing methods, nonlinear wave modulation method has high sensitivity assured through the interactions of crack surfaces and it is appropriate for fine crack detection [9,10]. Wave modulation is a phenomenon in which frequency components occur at sidebands, which are the frequencies corresponding to the sum and difference of two incident frequencies when waves having two different frequencies propagates through the sample, as shown in Figure 1 [11,12]. Microscopic damage, such as a fatigue crack in the specimen, causes peak generation at the sum and difference of the two input frequencies [13,14]. The occurrence of ultrasonic modulation is explained by material nonlinearity, hysteresis, and crack breathing, and such modulation is less affected by the measurement system than other nonlinear phenomena, because it only appears when there is interaction between two waves that have different frequencies. It is also more accurate than other nonlinear ultrasound methods, because it is unaffected by temperature change.

The nonlinear wave modulation technique was firstly introduced in US patents, and it has been continuously developed for crack detection [15]. An experiment performed on a concrete beam showed that the modulation of a high-frequency wave sharply increased in the presence of cracks [16]. Duffour et al. studied crack detection in a mild steel member, using an impact hammer and a lead zirconate titanate transducer (PZT) for low- and high-frequency (LF and HF) excitations, respectively [17]. Sohn et al. diagnosed an aircraft fitting-lug mock-up specimen, using a modified wave modulation technique with various input frequency combinations [18,19]. Kawashima explained the cause of nonlinear ultrasonic generation, using the stress–strain relationship, and its dependence on crack width [20]. The concept of contact acoustic nonlinearity (CAN) has been reported as explaining wave harmonics and modulation, in that these phenomena stem from the interactions of crack surfaces [21]. Sutin and Johnson suggested that changes to the transmission of HF signals were caused by changes in crack width during LF wave propagation, resulting in nonlinearities [22]. Zaitsev et al. [23] presented the wave modulation test results with pristine and cracked specimens, concluding that it is possible to detect micro-cracks with this method. Nonlinear ultrasonic modulation methods often require postprocessing steps for analysis. Lim et al. [24] proposed a method for detecting cracks using outlier analysis based on several test results. There have also been studies that have introduced a nonlinearity index (NI) and used this for crack detection. Lim et al. [25] conducted a non-destructive test with the sign of two factors: the skewness and the median of the NI distribution. Li et al. observed the correlation between the acoustic nonlinearity parameter of the stainless steel plate and the thermal-induced degradation using a phase reversal approach [26]. Rucka suggested an effective monitoring method of bolted joints using Lamb waves from a network of PZTs [27].

There have also been numerical nonlinear ultrasound modulation studies. Kawashima et al. [20] conducted numerical analyses with a perfectly flat and parallel crack model to observe changes to the harmonic component amounts. Lim et al. [28] modeled the crack by deleting one layer of the element and used it for crack detection analysis with wave propagation simulations. Liu et al. [29,30] analyzed changes of the PZT-induced Lamb wave passing through the defect numerically. Guan et al. numerically identified the direction of the micro-crack with the change of nonlinearity parameter [31]. Li et al. numerically investigated the second harmonic amount change from the Lamb wave passing through a micro-crack in the adhesive layer [32].

As described above, non-destructive evaluation using nonlinear wave modulation has been developed theoretically, experimentally, and numerically. However, as the amount of the modulated wave is affected by various variables, diagnosis with only one test is difficult. Therefore, it is necessary to develop a method that comprehensively analyzes the amount and frequency of modulated waves from repeated test results. In this study, the wave modulations due to seam cracks of a constant shape were analyzed, and the excitation conditions suitable for the test were presented. A numerical analysis technique consisting of three steps was proposed, and the modulated wave caused by cracking occurred as in the experiment. As a result, the crack detection algorithm based on the frequency of modulated waves found both seam cracks and fatigue cracks accurately.

## 2. Materials and Methods

### 2.1. Generation of Two Types of Crack Specimen

**Figure 2:**
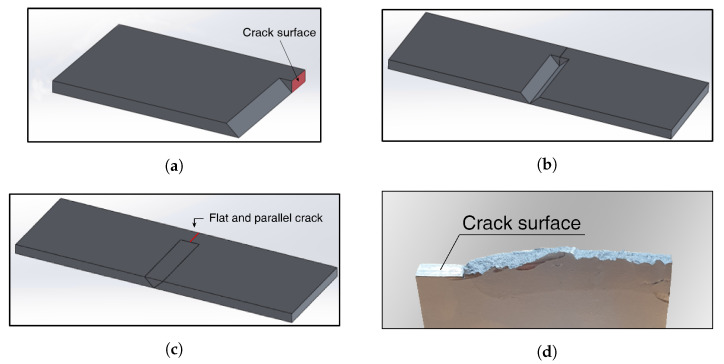
Fabrication of an aluminum specimen incorporating a seam crack: (**a**) construction of half of the specimen containing a flat crack surface, (**b**) arrangement of two parts, (**c**) specimen completion by grinding down the weld protrusion after welding, (**d**) crack surface observed in fractured specimen.

**Figure 3:**
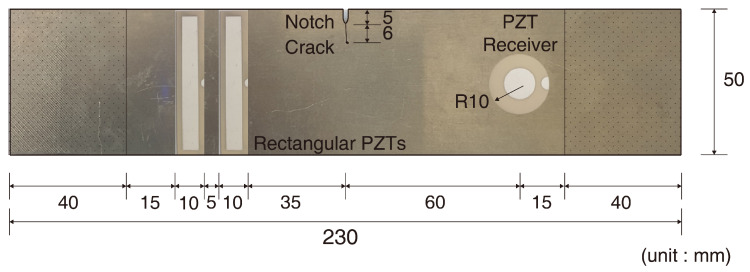
Geometry of aluminum specimen used in fatigue crack detection experiments. Both 40 mm long sections were fixed to the UTM, and repeated loads were applied to generate fatigue crack. Sinusoidal waves are incident by using two rectangular lead zirconate titanate transducers (PZTs), and a signal was measured by a circular PZT.

The width of the fatigue crack tends to decrease as it approaches the crack tip. The crack width is not constant due to the plastic deformation and fragmentation occurring during the crack propagation. Moreover, the propagation direction is irregular due to the influence of the grain boundaries. The fatigue crack is not suitable for quantitative analysis because of these irregularities. Before testing on fatigue cracks, we conducted a wave modulation test using specimens containing parallel and flat cracks for the quantitative analysis. Fatigue cracks have varying widths and irregular shapes, while artificial cracks have a uniform width. Figure 2 illustrates our specimen production process, drawn using Solidworks 2014 software (Dassault Systemes, Waltham, MA, USA). The specimen was made of aluminum 6061 (Daehan Corp., Daejeon, Korea), with a density of 2700 kg/m${}^{3}$, an elastic modulus of 70 GPa, and a Poisson’s ratio of 0.33. The size of the specimen and the position of the PZT wafers are the same as the fatigue specimen in Figure 3. However, there is no notch in the artificial crack specimen. First, the shape of the specimen in Figure 2a was manufactured using a machining process. Then, two symmetrical specimens were arranged together, and a v-shaped groove was formed at the center, as shown in Figure 2b, which was then filled by welding. An aluminum filler was used, to prevent the occurrence of material nonlinearity during experiments. After welding, excess weld was ground away, to make a rectangular specimen incorporating a seam crack, as shown in Figure 2c. The length of the seam crack could be controlled by adjusting the shape shown in Figure 2a.

The fatigue cracks were produced for the verification of the accuracy of the crack detection algorithm. Figure 3 represents the size of the specimen used in the experiment and the location of the PZT patches. The specimen was made of aluminum 6061, the same as the artificial crack specimen. The notch was placed in the middle of the specimen to generate fatigue cracks by concentrating the stress. A fatigue crack was produced by applying a tensile force of 0.7–7 kN at a frequency of 10 Hz after testing with specimen in the intact state.

**Figure 4:**
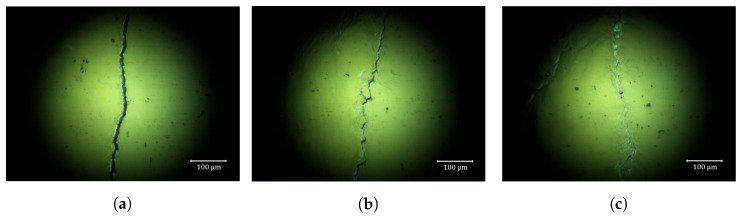
Photomicrographs of fatigue cracks in aluminum specimens, taken using an optical microscope. The photomicrographs were taken (**a**) near the notch, (**b**) in the middle of the crack, and (**c**) near the crack tip.

A photomicrograph of a fatigue crack in an aluminum specimen is presented in Figure 4. The crack images were taken using a Model HT004 microscope (HiMax Tech. Co. Ltd., Tainan, Taiwan) to capture the cracks of widths on the micro- or nanoscale. The surface of the specimen was chemically polished by removing the stain on the surface using a product called “Pikal care” to obtain clear images. It was found that, in contrast to the seam cracks, the widths of fatigue cracks are not uniform, rather changing significantly, dependent on the distance from the crack tip. Crack widths were <20 μm. The crack surfaces touch each other near the crack tip, and a clear and relatively wide crack can be seen in Figure 4a. Unlike the artificial cracks, fatigue cracks exhibit stripes, with their shape and size depending on the loading conditions and specimen materials [33]. The plastic deformation and fragmentation occurring near the crack tip during fatigue crack propagation creates the irregular shape of a fatigue crack. Figure 4b is a picture taken in the middle of the crack, and it can be seen that both crack surfaces have a similar shape, while not matching each other perfectly.

### 2.2. Excitation and Sensing Equipment

In this study, Lamb waves formulated by extension and reduction of PZTs were used, and, because the Lamb waves passed through all the cross sections of the sample, any internal sample defects could be detected. Waves incident to a specimen which has upper and lower free-boundary surfaces are reflected continuously, due to the difference in acoustic impedance between the medium and the air. Lamb waves generated by combining waves traveling in different paths can be processed for up to several meters, which has the advantage of testing a relatively broader area, in a non-destructive evaluation [34]. There are several wave generator types that can produce Lamb waves, such as point-source generators, piezoelectric transducers, and lasers [35,36]. The cost of PZT wafers are low, and they are easy to mount onto the surfaces of samples or structures [37]. It is also possible to monitor structure safety by receiving signals continuously over time, using a single installation.

The circular and rectangular piezoelectric wafers were made by APC International with APC 850 piezoelectric material (Manufacturer: APC International, Ltd., Mackeyville, PA, USA) because the characteristics of the APC 850 material make it suitable for both sensing and actuating. The rectangular PZT wafer was used for excitation, whereas the circular PZT was used for sensing. Rectangular PZT wafers with the same width as the specimen were used for the excitation to induce a relatively simple vibration mode compared to the circular PZT excitation. The PZT wafers were 0.5 mm thick and were polarized in the thickness direction. The material properties of the APC 850 material are listed in Table 1 [38]. The top surface of the PZT transducer is the anode, and the bottom is the cathode. A small part of the PZT’s upper surface was designed to be connected to the cathode for easy soldering and attachment. PZTs were attached to the surface of the aluminum specimen using Loctite 401 cyanoacrylate adhesives (Manufacturer: Loctite Corporation, Dublin, Ireland).

For the rectangular PZT made using APC850 piezoelectric material, an amplitude of ~3.5 nm was induced when 1 V was applied, although a smaller amplitude was observed when the PZT was attached to the plate, due to the stiffness of the plate. A relatively large amplitude is required when detecting cracks using wave propagation rather than stationary vibration. Experiments were performed using a National Instruments (NI) PXI data acquisition (DAQ) system (PXIe-1082, National Instrument Co., Austin, TX, USA). Two PZTs were controlled at different frequencies, using two 16-bit arbitrary waveform generators (AWGs, NI PXI-5421, National Instrument Co., Austin, TX, USA), and the response was measured at the sampling frequency of 1 MHz, using a high-speed digitizer (DIG, NI PXI-5122, National Instrument Co., Austin, TX, USA). The AWG and DIG were controlled simultaneously, using LabVIEW 2015 software (National Instruments, Austin, TX, USA).

The effects of excitation intensity, excitation duration, crack length, and excitation frequency can be observed by applying two waves with different frequencies to the artificial crack specimen. First, wave modulation tests were performed with varying HF and LF amplitudes, using aluminum plate specimens containing 0 (intact), 5, 10, and 20 mm flat cracks. The excitation frequencies used in the tests for each specimen are listed in Table 2. The resonance frequencies were selected from the frequency response function (FRF) obtained through the chirp excitation.

## 3. Quantitative Analysis of Wave Modulation with Seam Crack

The nonlinear parameter β is inversely proportional to the excitation amplitudes and wavenumbers of two incident waves as shown in Equations (1) and (2):

(1) $$\beta_{\mathrm{HF}-\mathrm{LF}}\approx \frac{M_{\mathrm{HF}-\mathrm{LF}}}{A_{\mathrm{LF}}A_{\mathrm{HF}}\kappa_{\mathrm{LF}}\kappa_{\mathrm{HF}}},$$

and

(2) $$\beta_{\mathrm{HF}+\mathrm{LF}}\approx \frac{M_{\mathrm{HF}+\mathrm{LF}}}{A_{\mathrm{LF}}A_{\mathrm{HF}}\kappa_{\mathrm{LF}}\kappa_{\mathrm{HF}}},$$

where $\beta_{\mathrm{HF}-\mathrm{LF}}$ and $\beta_{\mathrm{HF}+\mathrm{LF}}$ are modulated material nonlinear parameters; $\kappa_{\mathrm{LF}}$ and $\kappa_{\mathrm{HF}}$ are wavenumbers of two incident waves; and $A_{\mathrm{LF}}$ and $A_{\mathrm{HF}}$ are amplitudes of two incident waves, respectively [39,40]. The nonlinear parameters $\beta_{\mathrm{HF}-\mathrm{LF}}$ and $\beta_{\mathrm{HF}+\mathrm{LF}}$ are constants corresponding to sideband frequencies (HF-LF) and (HF+LF), respectively. Therefore, the magnitudes do not change, but the amount of the sideband increases according to the intensity of the LF and HF excitations.

**Figure 5:**
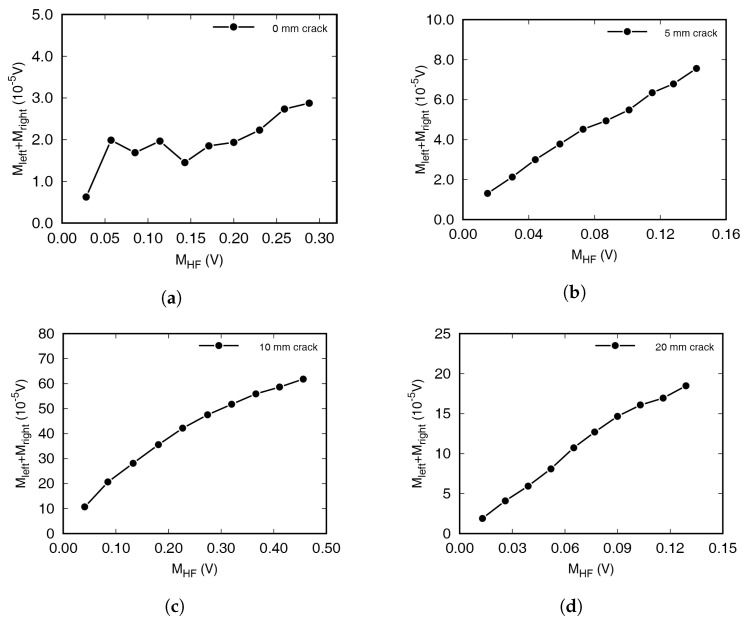
Effects of high-frequency (HF) wave amplitude on modulated wave generation. Specimens had crack lengths of (**a**) 0 mm, (**b**) 5 mm, (**c**) 10 mm, and (**d**) 20 mm.

**Figure 6:**
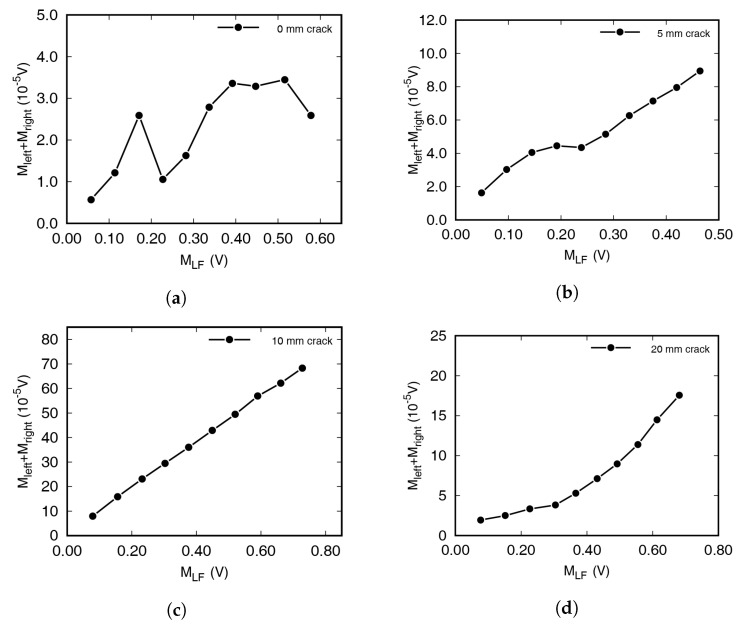
Effects of low frequency wave amplitude on modulated wave generation. Specimens had crack lengths of (**a**) 0 mm, (**b**) 5 mm, (**c**) 10 mm, and (**d**) 20 mm.

The amount of modulated wave increased in proportion to the LF and HF amplitude with damaged specimens. The linear tendency was observed as the intensity of several variables is the only factor that is not affected by the dynamic characteristics of the specimen. The magnitudes in the frequency spectra at the frequencies of incident waves and sidebands have been summarized in Figure 5 and Figure 6. Figure 5 shows the FFT magnitudes at both sidebands when increasing the HF amplitude from 0.1 to 1 V, in 0.1 V increments. The amplitude of the LF excitation was 1 V. In the figure, *M* represents the magnitude of the frequency spectrum. $M_{\mathrm{left}}$ and $M_{\mathrm{right}}$ represent the FFT magnitude at the left and right first sideband frequencies, respectively. The size of the LF signal, $M_{\mathrm{LF}}$, was almost constant, regardless of the amplitude of the HF signal, although it differed depending on the specimen. $M_{\mathrm{HF}}$ was directly proportional to the excitation amplitude, although the waves were stabilized for 1 s. Damaged specimens containing cracks showed a strong positive correlation, with coefficients > 0.97. For intact specimens, it is difficult to find clear correlations between the two axes, while on the other hand, strong positive correlations were clearly observable in Figure 5b–d. In Figure 6, on the other hand, the experimental results achieved by increasing the amplitude of LF—from 0.1 to 1 V, in 0.1 V increments, and with fixed HF excitation amplitude—have been summarized. As with the previous experimental results, the damaged specimens showed strong positive correlations between $M_{\mathrm{LF}}$ and the magnitude of the modulated waves, while the intact specimens (0 mm) showed no specific correlation.

In Figure 5 and Figure 6, the amount of modulated waves was largest with a 10 mm crack. It was expected that the amount of wave modulation increases as the length of a crack with a constant width increases. Although specimens with different crack lengths were produced in the similar way, the crack widths were only slightly different. In addition, the size of the resonance frequency and the frequency response vary depending on the crack length, and therefore no correlation between crack length and modulation wave generation could be detected experimentally.

As the excitation time increases, the incident wave interferes with the reflected wave and converges to stationary vibration. It is possible to induce crack opening and closing with a relatively small excitation amplitude using stationary vibration. During the wave convergence, the interval at which the modulated wave occurs is investigated. Crack-induced nonlinearity sensitively reacts to vibration mode, whereas material nonlinearity is less affected by vibration mode because it is distributed. Therefore, by repeating the test with stationary vibration for various frequency combinations, the effect of material nonlinearity can be investigated in the case with the least nonlinearity.

**Figure 7:**
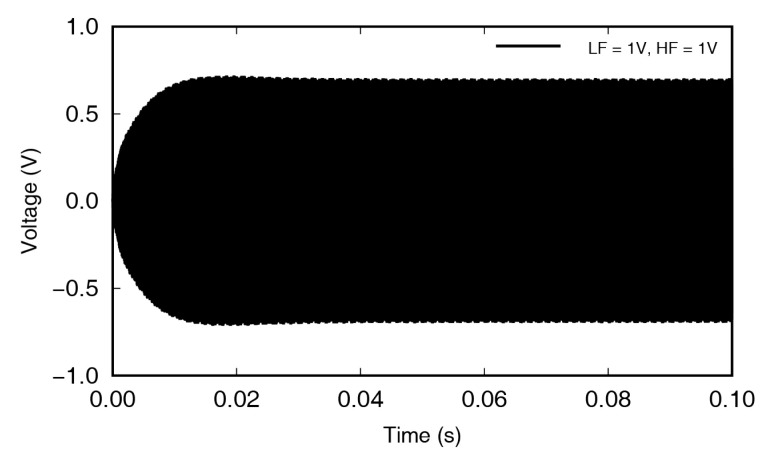
Time-domain signal measured using a circular PZT while the specimen was excited by low and high frequencies of 1 V.

Figure 7 shows the voltage signal, by circular PZT, with an LF excitation of 34.35 kHz and an HF excitation of 195.7 kHz, at 1 V amplitude. The strain on the aluminum specimen surface caused by the traveling wave was measured in the form of a voltage signal, through the circular PZT sensor. In experiments, the frequencies of excitation were selected based on the frequency response function (FRF) that was the result of chirp signal excitation, whose frequency gradually increased over time. In general, frequencies with large FRFs induces active specimen motion. However, large movements at the measuring position did not guarantee that the crack was breathing.

**Figure 8:**
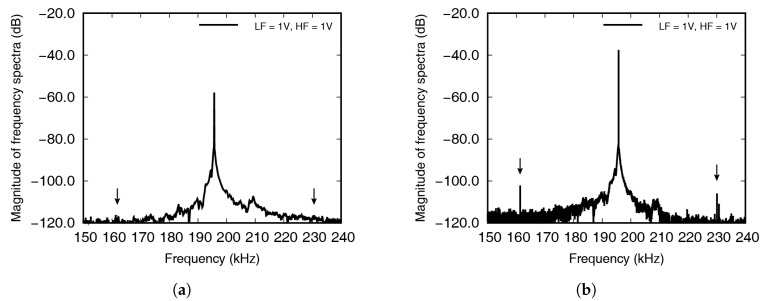
A Fourier-transformed signals, measured up to (**a**) 10 ms and (**b**) 100 ms, by circular PZT. The high frequency and both sideband frequencies are magnified.

It can be seen that the signal in Figure 7 gradually increased, up to 10 ms, and then showed almost constant amplitude after 20 ms. The incident waves were stabilized by interfering with the reflected waves. The Fourier transform of the results shown in Figure 7 is shown in Figure 8, where the frequency range near the high frequency has been enlarged, to show the occurrence of wave modulation more clearly. The 1st sideband frequencies have been indicated by the black arrows at 161.35 kHz and 230.05 kHz, respectively.

The Fourier transformed signal measured in the experiment is represented in Figure 8, with the result measured up to 10 ms shown in Figure 8a, and the result measured up to 100 ms in the frequency domain shown in Figure 8b. In Figure 8a, the modulated wave cannot be seen, while clear peaks are visible in Figure 8b, which was because the incident waves were stabilized after 10 ms, leading to the crack breathing. If the excitation energy is unlimited, it is advantageous to carry out testing until wave stabilization, as this will induce larger movements. In the experiment, the averaged result from ten repetitions was saved, to reduce the influence of noise, and in each test, the noise fluctuated, while the incident and modulated waves maintained a certain level. This meant that averaging the results of repeated tests decreased the amount of noise.

**Figure 9:**
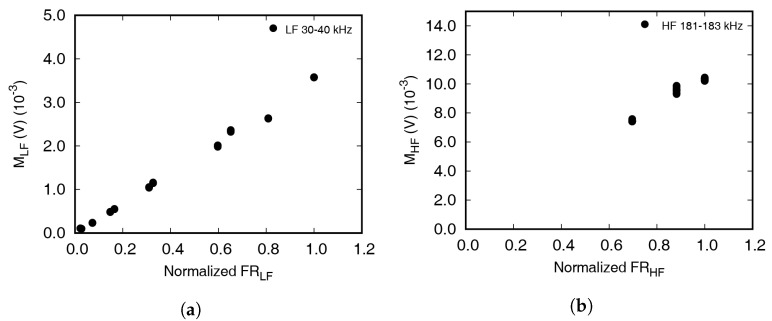
Correlation between the amplitude of frequency response function and the magnitude of frequency spectra at excitation frequencies. Results of (**a**) low frequencies from 30 to 40 kHz, and (**b**) high frequencies from 181 to 183 kHz with 1 kHz increments.

**Figure 10:**
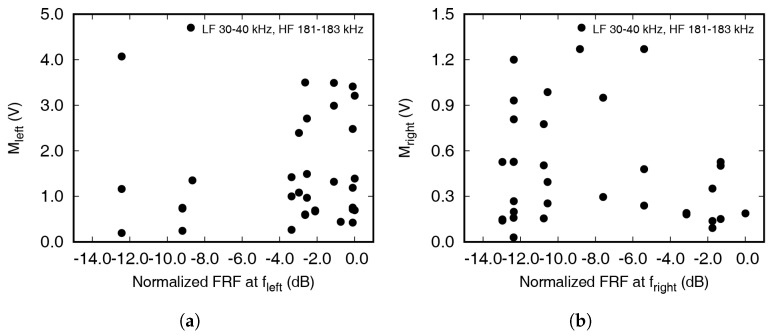
(**a**) Correlation between the amplitude of FRF and magnitude of frequency spectra at left 1st sideband (*f*_HF−LF_); (**b**) correlation between the amplitude of FRF and magnitude of frequency spectra at right 1st sideband (*f*_HF+LF_).

Figure 9 and Figure 10 represents the relationship between the amplitude of frequency response and the magnitude of frequency spectra. The experiments were performed with frequency combinations of low frequencies from 30 to 40 kHz and high frequencies from 181 to 183 kHz with 1 kHz increments. As mentioned above, $M_{\mathrm{left}}$, $M_{\mathrm{right}}$, $M_{\mathrm{LF}}$, and $M_{\mathrm{HF}}$ represent the FFT magnitude at the low excitation frequency, high excitation frequency, left first sideband frequency, and right first sideband frequency, respectively. FR represents the amplitude of the frequency response function at each frequency. As shown in Figure 9, the magnitudes in the frequency spectra of the excitation frequencies were proportional to the frequency response function obtained from the chirp signal excitation. Three points are closely located in Figure 9 as the experiment was repeated three times for one low excitation frequency. On the other hand, three groups of 11 points were observed in Figure 10 because the experiment was repeated 11 times, changing the low frequency for each high excitation frequency.

Figure 10a,b shows the effect of the FRF amplitude at each modulation frequency on the amount of modulated wave. If the FRF amplitude is large, the corresponding frequency wave is easily propagated and has a large value in the frequency domain. If modulated waves were generated and propagated at the crack surfaces, the correlation between the FRF amplitude and magnitude of frequency spectra should be observed. However, a certain tendency is not observed as the modulated frequency wave is not newly generated. The frequency shifts occurred due to partial transmission of the high frequency wave.

## 4. Numerical Simulations

The movements of the crack surface by Lamb wave propagation is quite complicated. In particular, the modes of the cracked surfaces due to stationary waves are difficult to predict. Realistic numerical analysis techniques that consider the movement of the PZT are introduced to investigate the behavior of a crack surface which is difficult to observe experimentally.

**Figure 11:**
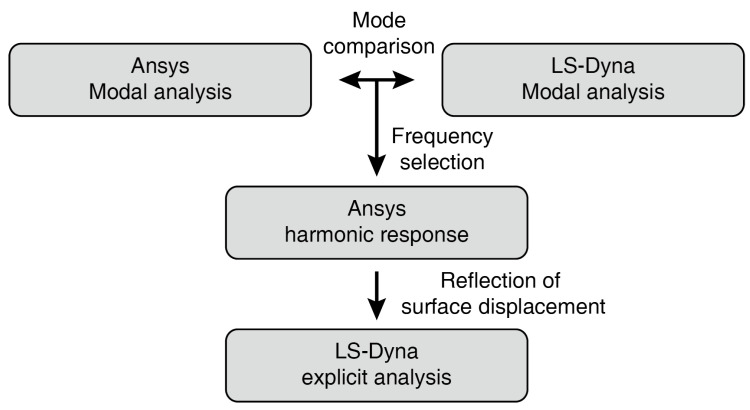
Flow chart for the numerical simulation of wave modulation in piezoelectric material.

Numerical analysis of PZT-induced Lamb waves with a seam crack is performed in three steps, as shown in Figure 11. Analysis of piezoelectric materials can be performed with Ansys, although the explicit analysis is unsupported, and transient analyses performed using Ansys require relatively long calculation times. However, LS-Dyna does not support piezoelectric material analysis; therefore, an efficient and reasonable analysis is performed by imposing the result achieved with Ansys software into LS-Dyna. Through modal analysis in Ansys, resonance frequencies and mode shapes for each frequency are obtained using an aluminum plate and attached PZT patches. Although numerical analyses are performed with the same geometry model, there are differences in the resonance frequencies, depending on the solvers. Modal analysis is therefore performed using LS-Dyna, to compare crack surface movements, and excitation frequencies at which cracks easily opened and closed are selected.

**Figure 12:**
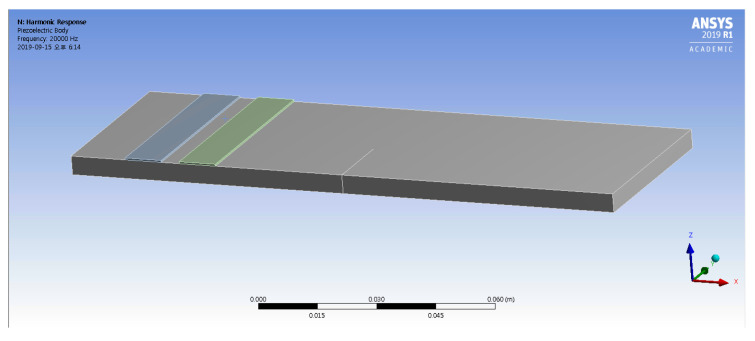
Geometry information for the numerical model created in Ansys. Two rectangular PZT patches are attached to the top surface of aluminum plate, and the seam crack can be seen in the middle of the specimen.

A schematic of the numerical aluminum plate model with two piezoelectric patches can be seen in Figure 12. The specimen is $150\times 50\times 5\;{\mathrm{mm}}^{3}$. The aluminum specimen and its PZT patches are modeled using the Solidworks three-dimensional modeling software, and the solid elements are generated using the Hypermesh commercial program based on the imported geometry model [41]. In this study, the four bottom surface vertices of the plate are fixed in all directions, bonded contact conditions are applied between the PZT patches and the aluminum plate, and a frictionless contact condition is applied between the crack surfaces. The characteristics of aluminum 6061 (density = 2700 kg/m${}^{3}$, Young’s modulus = 70 GPa) are used for the numerical simulation.

**Figure 13:**
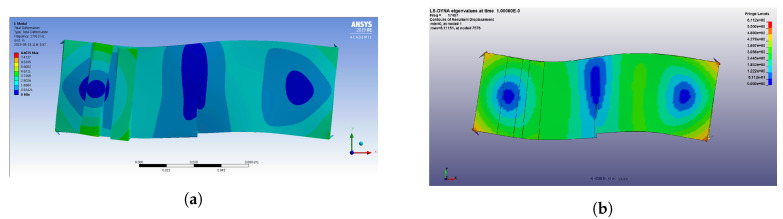
(**a**) Resultant displacement of modal analysis in Ansys at 17,910 kHz, and (**b**) numerical result of modal analysis using LS-Dyna.

The results of modal analysis in Ansys are shown in Figure 13a. Among the various modal frequencies, 17.910 kHz is chosen as the frequency of excitation to induce crack opening and closing. Figure 13b shows the modal analysis result achieved using Ls-Dyna, illustrating the similarity to the mode shape achieved using Ansys at 17.852 kHz. The LF wave makes cracks open and close, resulting in the truncation of the HF wave that is observed as a modulated wave in the frequency domain. The LF is selected using modal analysis, while the HF frequency was selected empirically. The HF and modulated wave frequencies should not collocate with integer multiples of the LF. If the modulated wave frequency is an integer multiple of the LF, it becomes difficult to distinguish the harmonic and modulated LF waves in the frequency domain. In the process of selecting excitation frequencies, the sampling frequency must be considered.

Next, harmonic response analyses are performed for 17.910 kHz and 183 kHz. The voltage of the lower surface of the rectangular PZTs is set to zero, and a sinusoidal electric boundary condition is applied to the PZT upper surfaces. It is assumed that the PZT patches and aluminum plate are strongly bonded together, which allows the displacement of the lower PZT patch surface to be extracted in each direction, for explicit analysis using Ls-Dyna. This process is repeated by exciting the PZT on the left, at 183 kHz. Explicit analysis is performed in Ls-Dyna, using the same numerical model as in Ansys. The direction and amplitude of each node are calculated from the extracted data, with directional information imposed by the defining vectors. Each node on the bottom of the PZT oscillates in an amplitude equal to the nodal displacement in the defined vector direction.

**Figure 14:**
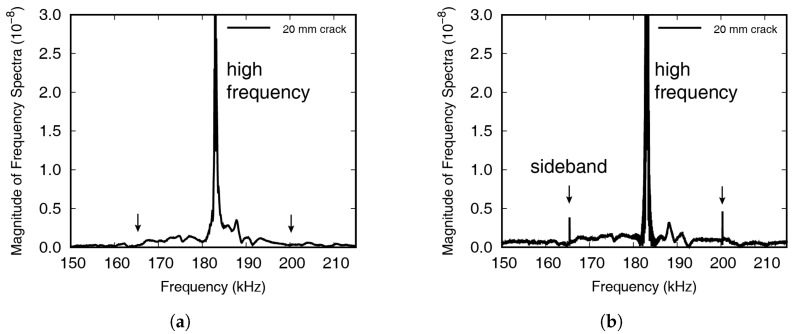
A Fourier-transformed signal in numerical simulation, measured up to (**a**) 10 ms and (**b**) 100 ms, at the node located on the center of circular PZT. The high frequency and both sideband frequencies are magnified.

**Figure 15:**
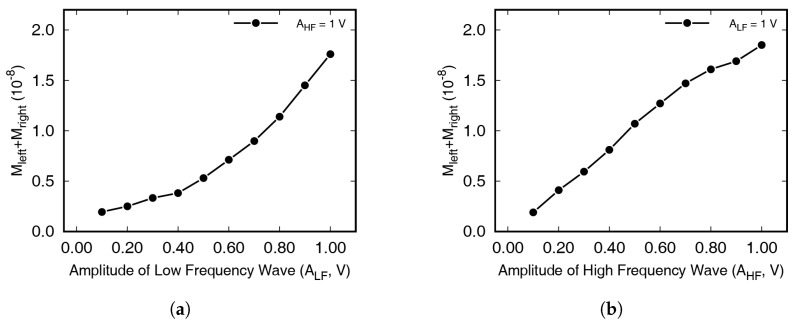
The amount of modulated wave generation in numerical analysis when a Lamb wave passes through a seam crack: (**a**) effect of LF amplitude, with constant HF wave amplitude; (**b**) effect of HF amplitude, with constant LF wave amplitude.

Numerical results for the nonlinear wave modulation test, achieved using the method introduced in the previous section, can be seen in Figure 14. Figure 14a represents the Fourier transformed signal measured up to 10 ms, and the modulated wave cannot be seen. The Fourier transformed result measured up to 100 ms is shown in Figure 14b, and clear peaks are observed. In the numerical simulation, wave modulation phenomenon is observed as in the experiment. Furthermore, numerical results can be seen in Figure 15, with the magnitude change at the first sideband frequencies shown according to the input voltage. Here, it can be seen that the magnitude of the modulated wave increased proportionally to the magnitude of the LF and HF wave. The crack surfaces did not only move in the primary mode, as, unlike in the case of longitudinal wave propagation, the Lamb waves induced by the waves incident on the surface exhibited complex vibration motions.

## 5. Crack Detection Algorithm

### 5.1. Nonlinear Indexes

From the observations in previous experiments, the algorithm that comprehensively analyzes the test results with various excitation conditions to diagnose the damage is introduced. Before describing the algorithm, two nonlinear indexes are explained. With the basic wave modulation technique, the occurrence of sidebands in the frequency domain is a criterion used to determine the safety of a sample. However, sidebands are rarely observed, even when the sample is intact, due to material nonlinearity or internal pore. Depending on the frequency combination, sidebanding may not occur if the interaction of the crack surfaces is inactivated. Therefore, it is difficult to determine the damage of a specimen with a single test. As such, it is required to reiterate the test by varying excitation conditions and comprehensively analyzing the data to ensure test accuracy [42]. This section introduces two indices counted by comparing the amount of sideband with ambient noise, which are used for a safety diagnosis. These indices were initially introduced in [42]; however, an understanding of their meaning and application has improved since this study. Experimental results captured using just one frequency combination cannot guarantee structural safety. Thus, experiments are repeated using a variety of excitation frequency combinations, and these results are analyzed using this method, to identify any indication of damage. Conducting experiments with *n* low-frequencies and *m* high-frequencies will generate a total of m×n data.

Structural integrity is determined based on the damage index (DI) and the intactness index (II). The DI indicates the rate of occurrence of modulated waves, with respect to the total number of experiments, as shown in Equation (3):

(3) $$\mathrm{DI}=\frac{\mathrm{Number}\;\mathrm{of}\;\mathrm{first}\;\mathrm{sideband}\;\mathrm{occurrences}}{2\times \mathrm{total}\;\mathrm{number}\;\mathrm{of}\;\mathrm{experiments}}$$

The first sidebands are located on both sides of the HF, shifted by the amount of an LF. When the number of experiments is m×n, there are 2mn first sidebands, because two sideband components are observed in each test; therefore, 2 was included in the denominator of Equation (3).

**Figure 16:**
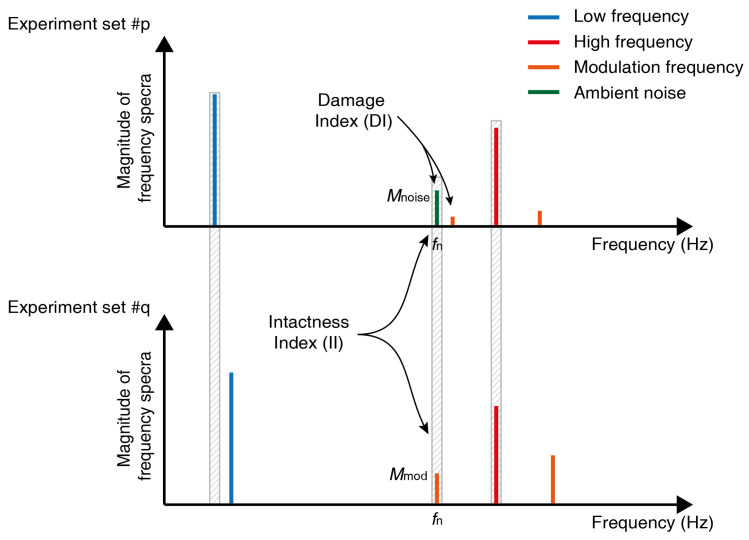
Comparison targets used to find a Damage Index (DI) and Intactness Index (II). The DI can be obtained by comparing the ambient noise with the magnitude of the modulated wave, in one experiment, while the II can be obtained by comparing magnitudes at one frequency, in two different experiments.

The II is an indicator of the occurrence rate of a phenomenon rarely seen in damaged specimens, while the DI represents the occurrence rate of a phenomenon frequently seen in damaged specimens. In experiments where wave modulation does not occur, a noise that is a local maximum can be found, and the frequency corresponding to this noise is called the $f_{n}$. In this example, an experimental result where the modulation frequency was located on the $f_{n}$ was found, and two experimental results have been compared, as shown in Figure 16. In the experiment where $f_{n}$ was the modulation frequency, the FFT magnitude at $f_{n}$ was designated as $M_{\mathrm{mod}}$, and the FFT magnitude at $f_{n}$ was designated as $M_{\mathrm{noise}}$ when $f_{n}$ was not the modulation frequency. Generally, $M_{\mathrm{mod}}$>$M_{\mathrm{noise}}$ for damaged specimens, while for in intact specimens, $M_{\mathrm{mod}}$ is often <$M_{\mathrm{noise}}$, in a phenomenon defined as (*). The DI2 and II2 can be calculated in the same way for the sum of both sideband sizes.

### 5.2. Excitation Conditions

For the testing, it is necessary to select the excitation frequency and excitation intensity. It is advantageous to use the resonance frequencies for efficient testing with less energy. However, it is difficult to use the resonance frequency every time from the viewpoint of continuous monitoring because the resonance frequencies are sensitive to environments such as temperature, boundaries, and external force. Moreover, it is not guaranteed that a frequency with a large amplitude on the FRF will induce the active movements of crack surfaces. Therefore, in this algorithm, suitable excitation intensities are selected for excitation frequencies in a certain section, and a comprehensively analyzing method is adopted. Modulation was rarely observed in either intact or damaged specimens when the excitation strength was insufficient. Although cracks existed in the specimen, safety diagnosis using the proposed nonlinear ultrasonic modulation technique could not produce an accurate result, indicating that an additional test—in which the excitation amplitude was varied—was needed, to check that the excitation strength used in the experiment was sufficient.

**Figure 17:**
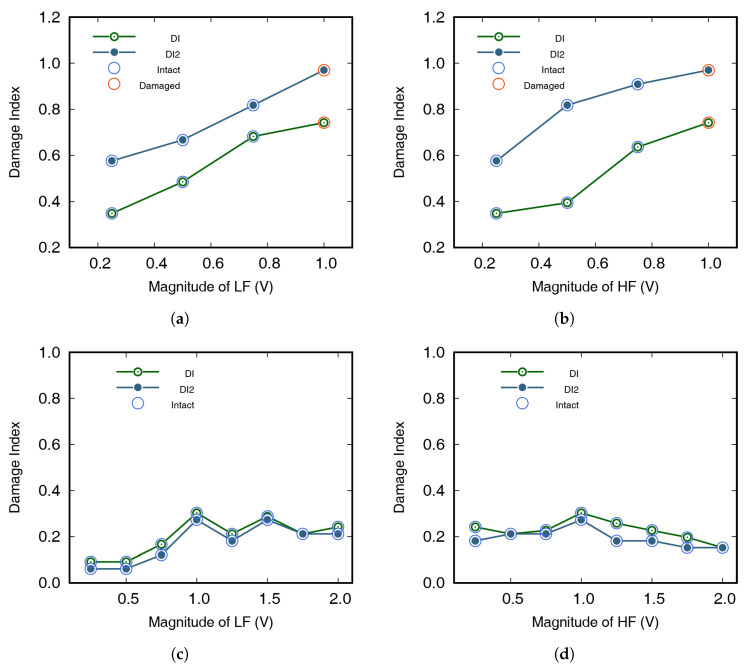
Damage index (DI) change with increasing excitation amplitude: (**a**) effect of LF amplitude on DI, with damaged specimens; (**b**) effect of HF amplitude on DI, with damaged specimens; (**c**) effect of LF amplitude on DI, with intact specimens; and (**d**) effect of HF amplitude on DI, with intact specimens.

Table 3 shows that, even if the excitation amplitude increased with the same frequency combination in the damaged sample, the modulated wave was still easily observed. As the excitation amplitude increased, the modulation wave could be seen, even at frequency combinations where no modulation was observed. Therefore, as the excitation of the damaged specimen increased, the DI increased steadily, which meant that, even if the test result from the suggested algorithm was that the specimen was intact, increasing the excitation could change the result to “damaged”, by increasing the DI, as shown in Figure 17a,b. The excitation amplitude used at the time of result change can be used for monitoring the system. However, in intact samples, the DI sometimes decreased without the change in results of the diagnosis, as shown in Table 4. If meaningful DI trends were not observed as excitation increased, the sample was considered to be intact, as shown in Figure 17c,d. In the first test, an appropriate excitation amplitude was selected by applying this method.

(4) $$\mathrm{II}=\frac{\mathrm{Number}\;\mathrm{of}\;(*)\;\mathrm{phenomenon}\;\mathrm{occurrences}}{2\times \mathrm{total}\;\mathrm{number}\;\mathrm{of}\;\mathrm{experiments}}$$

### 5.3. Damage Determination Criteria

The DI value is close to one when it has been derived from the experimental data with a damaged specimen. If DI = 1, this suggests that sidebands have been clearly observed for all frequency combinations, indicating the presence of a crack. If DI is < 1, this means wave modulation did not occur in some frequency combinations. In such cases, additional comparisons proceed using the II. Data obtained from intact specimens do not have a specific pattern. As such, the specimen requires frequent checking when analyzing the experimental results, to identify whether specific patterns are observed. The specimen may be categorized as an intact specimen, if such tendencies are not observed.

The size of the sideband may be smaller than the ambient noise, as the modulated wave may not occur for a frequency combination, even with a damaged specimen. However, noise values that are larger than those at sidebands in two different experiments are highly unlikely if there are sufficient excitation amplitudes. If the size of II is > threshold T, this means either the amplitudes of the input signals are insufficient, or the specimen may be intact. In this algorithm, if DI is < 1 and II is <T, the specimen is categorized as damaged. If DI is <1, if II is >T, the deciding test result is postponed. In this case, the algorithm is repeated for the sum of both sidebands (DI2 and II2) in order to exclude cases where the magnitude of one sideband is consistently negligible, while the other sideband occurs. The magnitudes at both sidebands show the difference as amplitudes at each frequency in the steady-state varies depending on the dynamic characteristic of the specimen [43]. Outlier analysis was also performed on $M_{\mathrm{noise}}$ to exclude signals from external electrical equipment. This analysis was conducted using (n−1) noise values with the exception of the signal at the modulated frequency, assuming that noise level fits an exponential distribution. Noise values outside the 99.99% confidence interval were treated as outliers. The algorithm for inspecting specimen defects using DI and II is summarized in Equation (5). $T_{1}$ and $T_{2}$ are thresholds for determining specimen damage and can be adjusted for each test environment. For the work described in this paper, $T_{1}$ and $T_{2}$ were set to zero.

(5) $$\mathrm{Test}\;\mathrm{result}=\left\{\begin{array}{cc} \mathrm{Damaged} & \mathrm{if}\;\mathrm{DI}=1\;\mathrm{or}\;\mathrm{II}<T_{1}\\ \mathrm{Damaged} & \mathrm{if}\;\mathrm{DI}2=1\;\mathrm{or}\;\mathrm{II}2<T_{2}\\ \mathrm{Intact} & \mathrm{if}\;\mathrm{DI}<1\;\&\;\mathrm{II}>T_{1}\;\&\;\mathrm{DI}2<1\;\&\;\mathrm{II}2>T_{2} \end{array}\right.$$

### 5.4. Application to Fatigue Crack

Relatively strong 4 V LF and 2 V HF were used as both boundaries of the specimen were strongly fixed. During excitation, LF from 30 to 40 kHz and HF from 181 to 183 kHz with a 1 kHz increments were used, with a total of 33 frequency combinations. This was proceeded by nonlinear wave modulation tests with the same excitation conditions. The results of these experiments with the three specimens are shown in Table 5. In the intact state, all three specimens were classified as intact as the DI and DI2 < 1, and II and II2 > 0. Following crack generation, specimens 1 and 3 appeared to have a crack as the DI was one, and specimen 2 was classified as damaged because the DI < 1, but II was zero. Based on these experiment results, we concluded that the safety of specimens could accurately be identified by the proposed defect diagnosis method.

## 6. Summary and Conclusions

Fatigue cracks are caused by repeated loads, although the applied stress is lower than the failure stress. Furthermore, it is difficult to predict failure timing accurately due to its growth characteristics. Early diagnosis is very important, as fatigue crack growth speed increases over time. Nonlinear ultrasonic methods are suitable for detecting small cracks because they use the interaction of crack surfaces instead of reflection and refraction. Among various nonlinear methods, it is known that the wave modulation technique has a distinct advantage of high sensitivity and being less affected by temperature change. In this study, wave modulation tests were performed with artificial seam crack that has constant width. The waves were generated using rectangular PZTs for simple-shaped waveforms, and the signals were measured at a point away from the crack and the excitation PZTs. It was found that excitation frequency is the most important among the variables, but movements of crack surfaces according to excitation frequency are difficult to expect. The excitation amplitude must be larger than a certain critical level. The excitation intensity could be determined by observing the change of index, as explained in this study, and the crack length was less affected than other factors as long as the crack width is uniform. A numerical simulation technique is developed to observe the crack motions. To simulate the movement of PZTs, the numerical procedure consists of three steps: modal analysis, harmonic response analysis, and explicit wave propagation analysis. In the numerical simulation, the change in amounts of modulated waves shows similar tendencies as in the experiments. From the quantitative analysis, suitable excitation conditions for the test and a crack diagnosis algorithm for comprehensive analysis with two nonlinear indexes were proposed. The two indexes were counted from the repeated test results, and the cracks were found with high accuracy. The proposed test methods and defects diagnosis algorithms were applied to the specimen before and after fatigue generation. For further quantitative analysis of modulated waves, the effects of different variables can be identified and optimized by the numerical analysis method suggested in this study. 

## Figures and Tables

**Table 1:** Piezoelectric properties of APC 850.

Property	Symbol	Value
Electromechanical coupling factor	$k_{p}$	0.63
	$k_{33}$	0.72
	$k_{31}$	0.36
	$k_{15}$	0.68
Piezoelectric charge constant (10${}^{-12}$ m/V)	$d_{33}$	400
	$d_{31}$	−175
	$d_{15}$	590
Poisson’s ratio	ν	0.35
Density ($\mathrm{kg}/m^{3}$)	ρ	7700
Elastic constant ($10^{10}$ ${\mathrm{Nm}}^{-2}$)	$c_{11}^{E}$	12.1
	$c_{12}^{E}$	7.54
	$c_{13}^{E}$	7.52
	$c_{33}^{E}$	11.1
	$c_{44}^{E}$	2.11
	$c_{66}^{E}$	2.26

**Table 2:** Excitation frequencies selected from the frequency response function (FRF) by applying a chirp signal excitation.

Crack Length (mm)	LF (kHz)	HF (kHz)
0	33.94	196.35
5	34.42	195.56
10	34.52	195.86
20	34.35	195.70

**Table 3:** DIs and IIs calculated by increasing the low-frequency (LF) and HF excitation on damaged specimens, using the proposed algorithm.

LF (V)	HF (V)	DI	DI2	II	II2	Test Result
0.25	1.0	0.348	0.576	0.394	0.212	Intact
0.5	1.0	0.485	0.667	0.318	0.182	Intact
0.75	1.0	0.682	0.818	0.121	0.030	Intact
1.0	1.0	0.742	0.970	0.015	0.0	Damaged
1.0	0.25	0.348	0.576	0.394	0.212	Intact
1.0	0.5	0.394	0.818	0.273	0.030	Intact
1.0	0.75	0.636	0.909	0.136	0.0	Intact
1.0	1.0	0.742	0.970	0.015	0.0	Damaged

**Table 4:** Calculated DIs and IIs achieved by increasing the LF and HF excitation on intact specimens, using the proposed algorithm.

LF (V)	HF (V)	DI	DI2	II	II2	Test Result
0.25	1.0	0.091	0.061	0.576	0.818	Intact
0.5	1.0	0.091	0.061	0.561	0.848	Intact
0.75	1.0	0.167	0.121	0.485	0.485	Intact
1.0	1.0	0.303	0.273	0.182	0.303	Intact
1.25	1.0	0.212	0.182	0.288	0.364	Intact
1.5	1.0	0.288	0.273	0.303	0.485	Intact
1.75	1.0	0.212	0.212	0.242	0.242	Intact
2.0	1.0	0.242	0.212	0.242	0.364	Intact
1.0	0.25	0.242	0.182	0.379	0.606	Intact
1.0	0.5	0.212	0.212	0.348	0.576	Intact
1.0	0.75	0.227	0.212	0.470	0.636	Intact
1.0	1.0	0.303	0.273	0.182	0.303	Intact
1.0	1.25	0.258	0.182	0.182	0.273	Intact
1.0	1.5	0.227	0.182	0.303	0.273	Intact
1.0	1.75	0.197	0.152	0.394	0.636	Intact
1.0	2.0	0.152	0.152	0.424	0.515	Intact

**Table 5:** Experimental results achieved by analyzing the test results from specimens with a real fatigue crack. Counted DIs and IIs were presented, and the integrity of the specimens were decided using these indexes. Indexes, which are the criteria for damage detection, are written in bold.

	Condition	Intactness Index	Damage Index	Test Result
Specimen #1	Intact	0.455, 0.515	0.348, 0.182	Intact
	Damaged	0	**1**	Crack
Specimen #2	Intact	0.061, 0.152	0.45, 0.242	Intact
	Damaged	**0**	0.97	Crack
Specimen #3	Intact	0.106, 0.060	0.621, 0.379	Intact
	Damaged	0	**1**	Crack
